# Biological Experimental Observations of an Unnoticed Chaos as Simulated by the Hindmarsh-Rose Model

**DOI:** 10.1371/journal.pone.0081759

**Published:** 2013-12-10

**Authors:** Huaguang Gu

**Affiliations:** School of Aerospace Engineering and Applied Mechanics, Tongji University, Shanghai, China; Georgia State University, United States of America

## Abstract

An unnoticed chaotic firing pattern, lying between period-1 and period-2 firing patterns, has received little attention over the past 20 years since it was first simulated in the Hindmarsh-Rose (HR) model. In the present study, the rat sciatic nerve model of chronic constriction injury (CCI) was used as an experimental neural pacemaker to investigate the transition regularities of spontaneous firing patterns. Chaotic firing lying between period-1 and period-2 firings was observed located in four bifurcation scenarios in different, isolated neural pacemakers. These bifurcation scenarios were induced by decreasing extracellular calcium concentrations. The behaviors after period-2 firing pattern in the four scenarios were period-doubling bifurcation not to chaos, period-doubling bifurcation to chaos, period-adding sequences with chaotic firings, and period-adding sequences with stochastic firings. The deterministic structure of the chaotic firing pattern was identified by the first return map of interspike intervals and a short-term prediction using nonlinear prediction. The experimental observations closely match those simulated in a two-dimensional parameter space using the HR model, providing strong evidences of the existence of chaotic firing lying between period-1 and period-2 firing patterns in the actual nervous system. The results also present relationships in the parameter space between this chaotic firing and other firing patterns, such as the chaotic firings that appear after period-2 firing pattern located within the well-known comb-shaped region, periodic firing patterns and stochastic firing patterns, as predicted by the HR model. We hope that this study can focus attention on and help to further the understanding of the unnoticed chaotic neural firing pattern.

## Introduction

It has been suggested that various complex oscillation patterns play important roles in excitable biological systems [1], [2]. With the help of nonlinear dynamics, the complex oscillations including chaos have been analyzed in depth. Chaos and bifurcations have been observed in actual nervous systems [3]–[6]. They have also been simulated in many theoretical neuronal models [7]–[24]. The Hindmarsh-Rose (HR) model has been one of the most commonly used models to identify the nonlinear dynamics of single neurons [12]–[24] and the spatiotemporal behaviors of neuronal networks [26]–[28]. The HR model is composed of three nonlinear, ordinary differential equations: 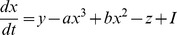
(1)

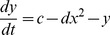
(2)

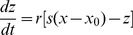
(3)


Here, the variable 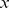 represents the membrane potential and 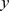 and 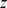 represent the recovery and the slow adaption current, respectively. The model has eight parameters: 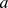, 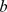, 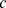, 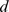, 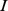, 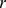, 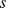 and 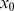. In this study, 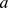 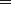
1, 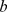 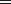
3, 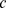 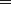
1, 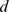 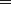
5, 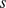 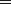
4 and 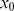 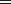
−1.6.

**Figure 1:**
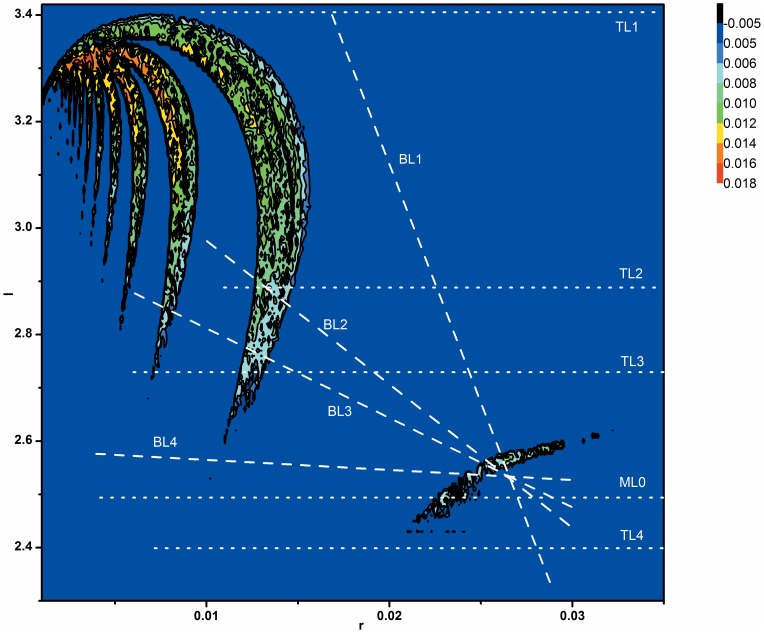
The largest Lyapunov exponent. The largest Lyapunov exponent in 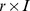 parameter space of the HR model (0.001
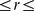
0.035, 2.3
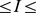
3.42). Colors shown in the right column are associated with the values of the largest Lyapunov exponent.

Recently, a comb-shaped chaotic region and dynamics, presented in multiple two-dimensional parameter spaces of the HR model, has received a lot of attention [12]–[25]. To clearly report the results from previous studies and to compare them with the biological experiments in the present study, the largest Lyapunov exponent in an 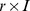 parameter space (0.001
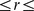
0.035 and 2.3
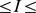
3.42) is reproduced, as shown in Fig. 1. The well-known, comb-shaped chaotic structure, located in the upper-left corner, is presented in yellow-orange, surrounded by a periodic region in blue. Notably, there is another chaotic region, separate from the comb-shaped chaotic one, located in the lower-right corner. The chaotic firing within this region was first identified, lying between period-1 and period-2 firings, by Fan and Holden in 1993 [12].

Chaotic behaviors within, and other firing patterns near, the comb-shaped region have been the focus of most of the previous investigations of the HR model. A period-doubling bifurcation cascade to chaotic firing [12]–[18] and chaotic firing following and lying in period-adding sequences [19]–[27] are similar to the behaviors seen along the thin dotted lines labeled TL2 and TL3 shown in Fig. 1. Period-doubling bifurcation that did not reach chaos and period-adding sequences without chaos was simulated, resembling the behaviors along lines TL1 and TL4, respectively. Bifurcation scenarios, similar to the three simulated in the HR model along lines TL1, TL2 and TL3, were observed in the biological experiments on a neural pacemaker [29]–[37]. Period-adding sequences with stochastic firings were observed in the biological experiments which were similar to the stochastic manner of the period-adding sequences without chaos simulated using the theoretical model [13], [14], [29], [30]. This was comparable to behaviors along line TL4. In the above-mentioned investigations, the chaotic firing pattern and region lying between period-1 and period-2 firings has not yet been identified and has not been taken into account.

The chaotic firing, simulated by Fan and Holden, lying between period-1 and period-2 firings and located within a bifurcation scenario, was obtained by decreasing 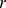 with fixed 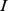 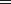
2.5 [12] and was similar to the behavior along the dotted line labeled ML0 in Fig. 1. The behavior after the period-2 firing appeared to be period-adding sequences without chaos (Fig. 6 in ref[12]). The chaotic region lying between period-1 and period-2 firings has recently been discovered in two other papers in figures similar to Fig. 1. In a study by González-Miranda, one chaotic region was found in the middle-right area (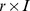
parameter space) of a figure (Fig. 6 in ref[19]) and other chaotic regions were found in the lower-right corner (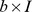 parameter space) in one figure (Fig. 1 in ref[24]) and in the lower-left corner (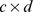
parameter space) of another figure (Fig. 4(c) in ref[24]) in a study by Rech. However, this chaotic region was not mentioned in either of these papers [12], [24]. Two examples of the chaotic firing lying between period-1 and period-2 firings within a bifurcation scenario have been mentioned in our previous experimental investigations on a neural pacemaker [31], [32]. One bifurcation scenario terminated at the period-2 firing due to decreasing extracellular calcium concentration [31]. This chaotic firing manifested characteristics different from those simulated in the HR model. The other scenario, from period-1 firing, to the chaotic firing, to period-2 firing, and to period-1 firing was induced by decreasing extracellular calcium concentration and by applying extracellular caesium [32]. No chaotic firing or stochastic firing appeared after the period-2 firing in either experimental scenario. These two experimental examples imply the existence of chaotic firing lying between period-1 and period-2 firings in an actual nervous system. The parameter space relationships between the chaotic firing lying between period-1 and period-2 firings and other previously identified chaotic, periodic and stochastic firing patterns remain unclear in both the HR model and in the experimental results.

Speculating from Fig. 1, the HR model presents at least four typical bifurcation scenarios containing the chaotic firing lying between period-1 and period-2 firings, akin to the behaviors along the lines BL1, BL2, BL3 and BL4. The initial part of the scenarios was chaotic firing lying between period-1 and period-2 firings. The following parts, after period-2 firing, were period-doubling bifurcation not to chaos, period-doubling bifurcation to chaos, period-adding sequences with chaos, and period-adding sequences without chaos, respectively. In this study, we provide fundamental, biological, experimental observations, in different isolated neural pacemakers, of four bifurcation scenarios containing the chaotic firing lying between period-1 and period-2 firings. This is accomplished by decreasing extra-cellular calcium concentration. Four experimental bifurcation scenarios and the chaotic firings lying between period-1 and period-2 firings closely matched those along the lines from BL1 to BL4, simulated using the HR model. The experimental results not only provide stronger evidence of the existence of chaotic firings lying between period-1 and period-2 firings than that reported in the previously two examples, but also present relationships in the parameter spaces between this chaotic firing and other chaotic, periodic and stochastic firing patterns in the real nervous system, as predicted by the HR model.

The rest of this paper is organized as follows. Simulation results are reproduced using the HR model in Section 2. Section 3 presents the experimental model. Experimental results are reported in Section 4. The conclusion and discussion are presented in Section 5.

## Materials and Methods

### Ethics Statement

All rats were treated in strict accord with institutional protocols. All experiments were approved by the university biomedical research ethics committee. All surgery was performed under sodium pentobarbital anesthesia (40 mg/kg, i.p.; supplemented as necessary), and all efforts were made to minimize suffering.

### Experimental model

Bennet and Xie developed an animal model of chronic constriction injury (CCI) of the rat sciatic nerve [38]. The CCI model appears to reproduce many features of neuropathic pain disorders such as spontaneous foot lifting, mechanical allodynia and heat hyperalgesia and is widely used in studies of neuropathic pain [38], [39]. Electrophysiological recordings from myelinated primary afferent axons revealed the spontaneous impulsive activity that originated at the site of nerve, which lead to abnormal spontaneous pain and also contribute to the maintenance of allodynia and hyperalgesia. In a series of previous investigations, the CCI model was used to investigate the bifurcations and chaos of spontaneous neural firing patterns [29]–[37]. In this context, the model was called an experimental neural pacemaker, which resembles a single neuron. This is the model used for the current study.

A surgical operation was performed to produce a neural pacemaker [30], [39]. Male Sprague Dawley rats, weighing 150–300 g, were injured using chronic ligatures. After 6–14 days, the injured site was exposed and perfused continuously with 34°C Kreb's solution in which the controlling extra-cellular calcium concentration ([Ca^2+^]_o_) was 1.2 mmol/L (mM). The spike trains from the spontaneous firing generated in the membrane of the injured site were recorded from the individual fibers that ended at the injured site, using a Powerlab system (Australia) with a sampling frequency of 10.0 kHz. The time intervals between the maximal values of the successive spikes were calculated in a series as ISIs. A neural pacemaker often generates period-1 firing pattern in the control condition. Several bifurcation scenarios beginning from period-1 firing, such as period-adding sequences with chaos, period-doubling bifurcation to chaos, period-adding sequences with stochastic firings, and bifurcation scenarios from period-1 firing to period-1 firing through a complex or simple process, have been observed in previous studies with decreasing [Ca^2+^]_o_ [29]–[37].

In the present study, [Ca^2+^]_o_ was chosen as the bifurcation parameter, based on its performance in a number of previous studies [29]–[37]. A neural pacemaker that could generate period-1 firing at the control condition was chosen and the solution was then replaced with 0 mM [Ca^2+^]_o_. Although the replacement is sudden, the effects on the membrane of the neural pacemaker, due to the change in ion concentrations, are gradual and continual. The membrane dynamics can be changed slowly enough to exhibit a transition procedure, but fast enough to reach a certain firing pattern, within a finite time span, that is different from the initial pattern. This type of gradual change in the biological parameter has been employed when exploring bifurcations of neural firing patterns in the present set of biological experiments on the neural pacemakers and on other biological tissues [40]–[43].

### Correspondence of the experimental and HR model parameters

Calcium-dependent potassium was identified in the neural pacemakers in a number of investigations [44]–[48]. The decrease of [Ca^2+^]_o_ can induce a decrease of inward Ca^2+^ influx and a decrease in the current of the calcium-dependent potassium channel, which can lead to the depolarization of the membrane potential. To a certain extent, the enhancement of membrane potential induced by the depolarization is equivalent to that induced by the increase of parameter 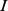 in the HR model. In addition, the decrease of Ca^2+^ influx also means less inward current. Compared with the inward sodium current, the decrease of the inward Ca^2+^ current was much smaller. The decrease of inward Ca^2+^ current can be neglected.

From the viewpoint of physiology, Ca^2+^ influx is a slow factor that is described by the variable 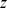 of the HR model. The effect of decreasing [Ca^2+^]_o_ in the experiment can be simulated through adjusting the parameters in the third equation, 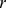, 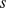 and 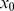. It is difficult to determine which parameter exactly corresponds to the decrease of Ca^2+^ influx because the HR model does not contain ionic channel. So we calculate bifurcations in the 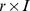, 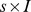 and 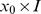 parameter spaces. The bifurcations observed in the experiment close match those simulated in the 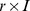 parameter space, while only a part of bifurcations observed in the experiment can be simulated in the 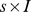 and 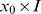 parameter space. Decreasing [Ca^2+^]_o_ in the experiment can produce an effect somewhat equivalent to that induced by decreasing 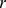 and increasing 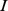 simultaneously. Therefore, the simulation result of bifurcations in the 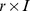 parameter space is adopted in this paper.

In general, the degree of decreasing 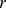 and increasing 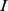 induced by decreasing [Ca^2+^]_o_ is different for different neural pacemakers. The difference in the degree is measured by a different value of slope in the equation composed of 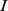 and 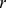. For example, four different slope values were chosen for the four lines (BL1 to BL4) corresponding to different neural pacemakers.

## Results

### The simulation results of the HR model

**Figure 2:**
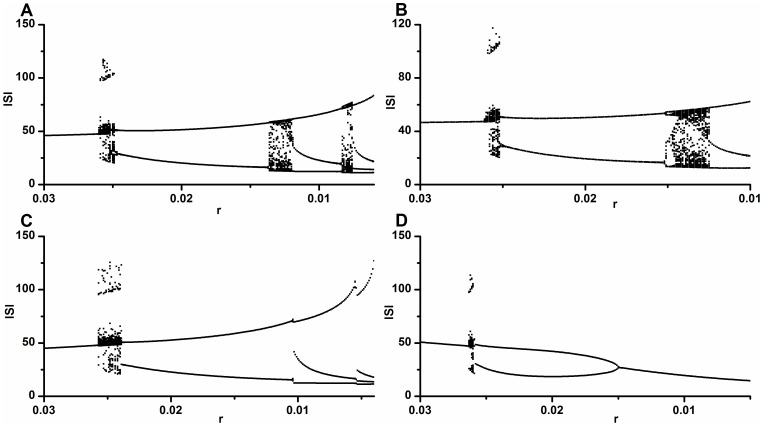
Bifurcation scenario in the HR model. Bifurcation scenario of the ISI series dependently with decreasing 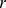
and increasing 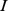. (A) 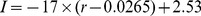; (B) 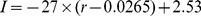; (C) 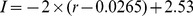; (D) 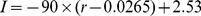.

The bifurcation scenarios along lines BL2, BL3, BL4 and BL1 are reproduced and compared with experimental results in this study. The equations for BL3, BL2, BL4 and BL1 are 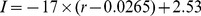, 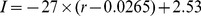, 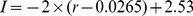, and 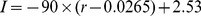, respectively. The four lines have different values for slope. The simulation results of bifurcation scenarios with decreasing 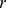, and increasing 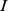, are shown in Fig. 2. The initial part of bifurcation scenarios is from period-1 firing, to chaotic firing, and to period-2 firing. After period-2 firing, the four bifurcation scenarios manifest different processes, outlined as follows.

The scenario along BL3 is period-adding sequences with chaos from chaotic firing, to period-3 firing, to chaotic firing, and to period-4 firing, as shown in Fig. 2(A) (0.006
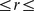
0.03). The bifurcation process along BL2 is period-doubling bifurcation to chaos from period-4 firing, to chaotic firing, and to period-3 firing, as depicted in Fig. 2(B) (0.01
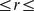
0.03). The scenario corresponding to BL4 is period-adding sequences without chaos from period-3 firing, to period-4 firing, as illustrated in Fig. 2(C) (0.004
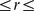
0.03). This scenario manifests a process similar to the one simulated by Fan and Holden [12]. The bifurcation scenario along line BL1 is from period-1 firing, to chaotic firing, to period-2 firing, and to period-1 firing, as shown in Fig. 2(D) (0.004
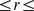
0.03). The scenario along line 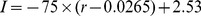 is from period-1 firing, to chaotic firing, to period-2 firing, to period-4 firing, to period-2 firing, and to period-1 firing (not shown). In the region right of the comb-shaped chaotic region and above the chaotic region lying between period-1 and period-2 firing, period-doubling bifurcations that cannot reach chaos appear after the chaotic firing lying between period-1 and period-2 firings. The bifurcation scenario along BL1 exhibits the simplest process in this region.

**Figure 3:**
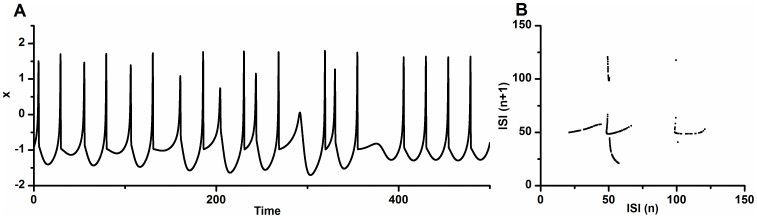
Chaotic firing in the HR model. Chaotic firing lying between period-1 firing and period-2 firing with 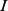 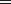
2.53 and 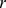 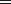
0.0245. (A) Spike trains; (B) The first return map of ISI series

The chaotic firing lying between period-1 firing and period-2 firings when 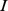 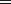
2.53 and 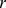 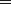
0.0245 in the HR model is shown in Fig. 3. The spike trains manifest an irregular characteristic, as shown in Fig. 3(A). There exist continual spikes with middle interspike intervals (ISIs), coupled spikes with a long ISI and a short ISI, and a spike with a much longer ISI. The first return map of the ISI series exhibits a deterministic structure, as depicted in Fig. 3(B).

### Overview of the experimental results

Spontaneous firings were recorded in 194 neural pacemakers in 47 rats, with decreasing [Ca^2+^]_o_ from 1.2 mM to 0 mM. One trial was performed with the experimental pacemaker. Only a little of neural pacemakers generate bifurcations from period-1 bursting to period-1 spiking. Most of the neural pacemakers generated bifurcation scenarios terminated at a certain firing before period-1 spiking. These bifurcations included period-doubling bifurcation not reaching chaos, period-doubling bifurcation to chaos, period-adding sequences with chaos, and period-adding sequences with stochastic firings. These were similar to those identified in previous investigations [29]–[37]. In these bifurcation scenarios, period-1 firing was changed into period-2 firing directly or via a stochastic firing. These bifurcations were also similar in appearance to the ones simulated along lines TL1, TL2, TL3 and TL4 using the HR model.

Most of bifurcations recorded in 194 neural pacemakers did not contain chaotic firing lying between period-1 and period-2 firings when [Ca^2+^]_o_ was decreased. Only 22 pacemakers generated bifurcations containing chaotic firing pattern lying between period-1 and period-2 firings. At 0 mM [Ca^2+^]_o_, bifurcations of one pacemaker reached period-1 firing, four pacemakers reached period-5 firing, two pacemakers reached chaotic firing after period-4 firing, seven pacemakers reached period-4 firing, three pacemakers reached chaotic firing after period-3 firing, and five pacemakers reached period-3 firing. Other than the 22 pacemakers, there existed a pacemaker that generated chaotic firing pattern lying between period-1 and period-2 firing patterns at the control condition.

Bifurcations of eleven, three, seven, and one pacemakers manifested bifurcations resembling those simulated using the HR model along the lines BL3, BL2, BL4, and BL1, respectively. The initial part of these bifurcation scenarios was chaotic firing lying between period-1 and period-2 firings. The behaviors after period-2 firing were period-doubling bifurcation not to chaos, period-adding sequences with chaos in firing patterns, period-doubling bifurcation to chaos in firing patterns, and period-adding sequences with stochastic firings, respectively.

### Experimental bifurcation scenarios

**Figure 4:**
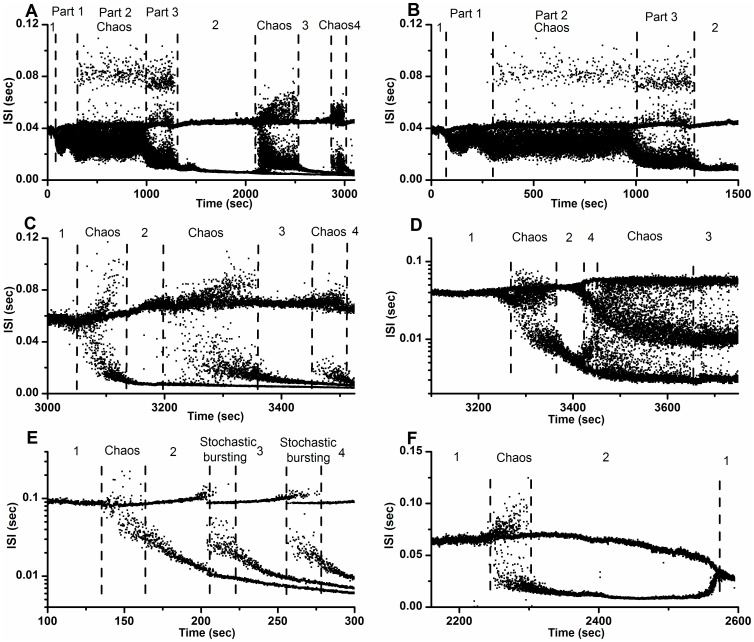
Bifurcation scenarios observed from different neural pacemakers with decreasing [Ca^2+^]_o_. The initial part was from period-1 firing, to chaotic firing, and to period-2 firing. Bifurcation scenario after period-2 firing was different. (A) From chaotic firing, to period-3 firing, to chaotic firing, to period-4 firing. The chaotic firing was divided into 3 parts; (B) A part of Fig. 4(a). Bifurcation scenario from period-1 firing to period-2 firing via chaotic firing; (C) From chaotic firing, to period-3 firing, to chaotic firing, to period-4 firing; (D) From period-4 firing, to chaotic firing, to period-3 firing; (E) From stochastic firing, to period-3 firing, to stochastic firing, to period-4 firing; (F) Period-1 firing.

In this case only two, one, one and one examples of bifurcation scenarios corresponded to the ones simulated using the HR model along lines BL3, BL2, BL4, and BL1, respectively (Fig. 4). The bold dashed vertical lines separate the different periodic and chaotic or stochastic firing patterns. The numbers represent the periods of the periodic firing patterns.

One example, resembling one produced by the HR model along line BL3, was shown in Fig. 4(A). The process was from period-1 firing, to chaotic firing, to period-2 firing, to chaotic firing, to period-3 firing, to chaotic firing, to period-4 firing. The transition from period-1 firing to period-2 firing via the chaotic firing is illustrated in Fig. 4(B). The chaotic firing lying between period-1 firing and period-2 firing can be divided into 3 parts: part 1 (between 80.1 sec and 273.4 sec), part 2 (between 273.4 sec and 988.7 sec) and part 3 (between 988.7 sec and 1300.6 sec). The thin dashed vertical lines separate the different parts. The other example was shown in Fig. 4(C). In this case the bifurcation process was also from period-1 firing, to chaotic-firing, to period-2 firing, to chaotic firing, to period-3 firing, to chaotic firing, and to period-4 firing. The chaotic firing lying between period-1 and period-2 firings exhibited a simpler pattern than the first example. These two examples show that period-adding sequences with chaos appeared after the chaotic firing lying between period-1 and period-2 firings, like the behaviors along line BL3.

The bifurcation scenario resembling the one simulated using the HR model along line BL2 was depicted in Fig. 4(D). The process was from period-1 firing, to chaotic-firing, to period-2 firing, to period-4 firing, to chaotic firing, and to period-3 firing. Period-doubling bifurcation to chaos can appear after chaotic firing lying between period-1 and period-2 firings, akin to the behaviors along line BL2.

A bifurcation scenario from period-1 firing, to chaotic firing, to period-2 firing, to stochastic firing, to period-3 firing, to stochastic firing, and to period-4 firing was shown in Fig. 4(E). Stochastic firings, such as the ones in this scenario, were identified as induced by noise at the period-adding bifurcation point within the period-adding sequences without chaos, like the behaviors along the line TL4. This bifurcation scenario shows that the period-adding sequences with stochastic firings appeared after chaos lying between period-1 and period-2 firings, like the stochastic behavior along line BL4.

A bifurcation scenario from period-1 firing, to chaotic firing, to period-2 firing, to period-1 firing was illustrated Fig. 4(F). This bifurcation scenario resembling the one simulated using the HR model along the line BL1, and also similar to the one identified in previous experiments, was induced by decreasing [Ca^2+^]_o_ and applying 2.5 mM extracellular caesium [32]. No examples similar to the one along line 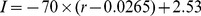 were observed in the experiments.

The behaviors after period-2 firings in the four bifurcation scenarios, manifested characteristics similar to the ones previously identified, that were investigated in both experimental and theoretical models, and that did not contain the chaotic firing lying between period-1 and period-2 firings [29]–[37]. The detailed characteristics of firing patterns after period-2 firing are not addressed in this study. The chaotic firings lying between period-1 and period-2 firings are analyzed in the following subsections.

### Spike trains and the first return map of the experimental chaotic firing

**Figure 5:**
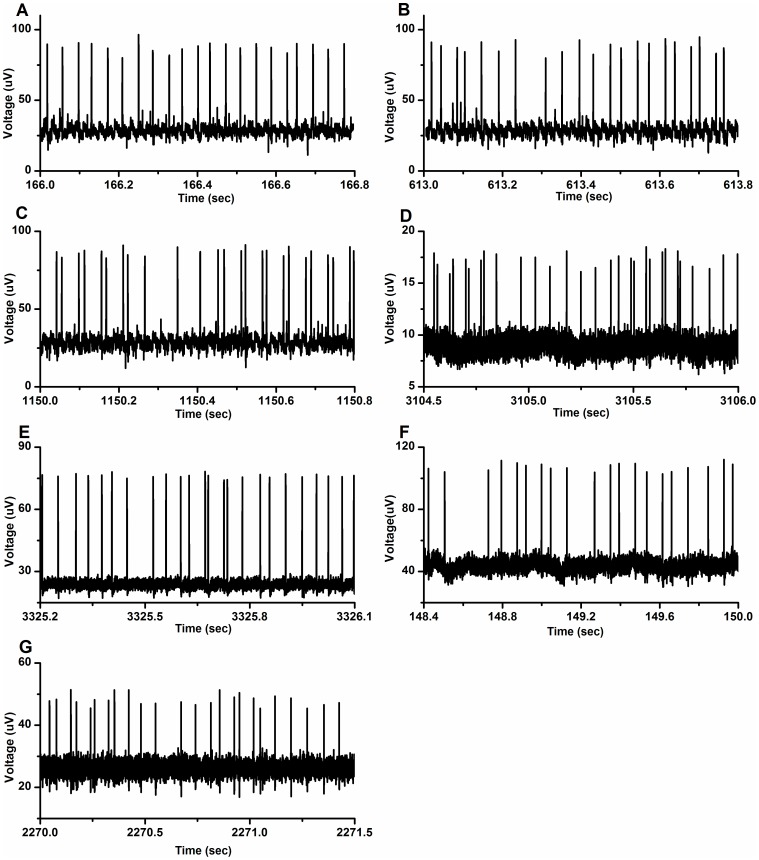
Spike trains observed from different neural pacemakers. Spike trains of the chaotic firing lying between period-1 and period-2 firings. (A) Part 1 of the first example; (B) Part 2 of the first example; (C) Part 3 of the first example; (D) The second example; (E) The third example; (F) The fourth example; (G) The fifth example.

The spike trains of the chaotic firing lying between period-1 and period-2 firings of the part 1, part 2 and part 3 of the first example, second example, third example, fourth example, and fifth example exhibited an irregular characteristic, as shown in Figs. 5(A)–5(G), respectively. Other than part 1 of the first example, the spike trains of the other six chaotic firings exhibited characteristics similar to that of the HR model (Fig. 3(A)). There were also continual spikes with middle ISIs, coupled spikes with a long ISI and a short ISI, and a spike with a much longer ISI in the spike trains. However, no longer ISIs were found in part 1 of the first example.

**Figure 6:**
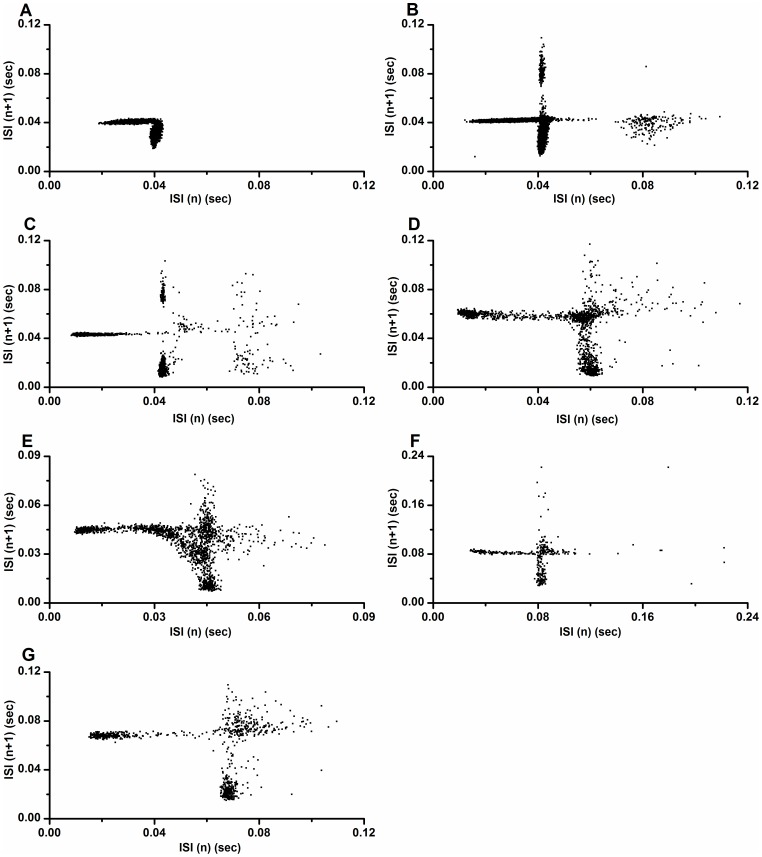
The first return map of the chaotic firing lying between period-1 and period-2 firings. The first return map of ISI series observed from different neural pacemakers. (A) Part 1 of the first example; (B) Part 2 of the first example; (C) Part 3 of the first example; (D) The second example; (E) The third example; (F) The fourth example; (G) The fifth example.

The first return maps of the ISI series of seven chaotic firings exhibit a deterministic structure as illustrated in Fig. 6. As shown in Fig. 6(A), the return map of part 1 of the first example is similar to the lower-left corner of that simulated using the HR model (Fig. 3(B)). This may have been because longer ISIs were not present in the first part of chaotic firing in first example. The other six return maps manifested structures similar to that of the HR model, as shown in Figs. 6(B)–(G).

### Deterministic characteristics of the experimental chaotic firing

Other than the first return map, we computed the normalized prediction error (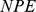) of ISI series to further estimate the deterministic property of the chaotic firing lying between period-1 firing and period-2 firings [30], [31], [37], [49], [50]. In this study, we used the simple nearest-neighbor method [49], [50] with the following algorithm:

A time series, 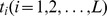, is transformed to 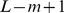 state points in space 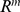 with a dimension 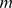. For a point 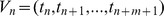 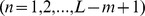 in space 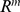, 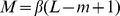 points nearest to 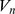 are chosen (0
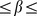
1) and written as 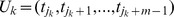 (1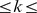 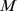). The average 
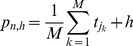
 is then used to approximate the future value 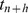. The difference 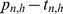 is the 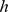-th step prediction error for point 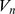. For all state points, the 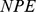 is defined as follows: 
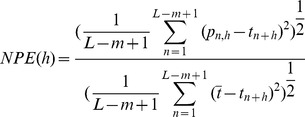
(4)


Where 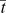 is the average of time series 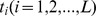. By definition, values of 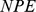 far below 1.0 indicate that the time series is predictable beyond the baseline prediction of the series mean. The 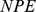 of raw data and surrogate data were compared. The surrogate data, generated by random shuffle method (shuffling the original sequence randomly) [51], showed the same probability distribution as the original data, but the deterministic temporal structures within the ISI series were broken. The 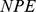 value of the original chaotic ISI series was much less than 1.0 in a short term of prediction and approached 1.0 in a long term, but the 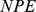 of the surrogate data was always nearly equal to 1.0, showing the deterministic property of the original series.

**Figure 7:**
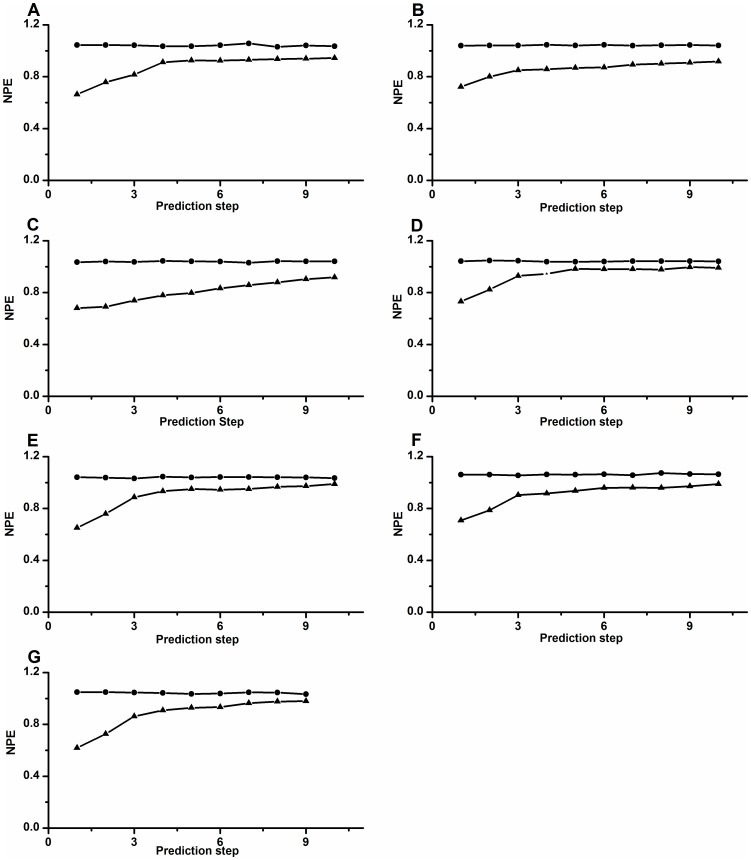
The 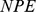. The 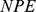 of chaotic firings lying between period-1 and period-2 firings observed from different neural pacemakers. Line with triangle, original data; line with circle, mean of 10 realizations of surrogate data. (A) Part 1 of the first example; (B) Part 2 of the first example; (C) Part 3 of the first example; (D) The second example; (E) The third example; (F) The fourth example; (G) The fifth example.

The 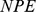 of the chaotic firing lying between period-1 and period-2 firings of seven examples were calculated with 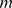 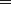
3–8 and 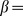
0.5%, 1%, and 2%, respectively. The 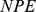, with increasing prediction step (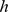), manifested a changing trend from a value much less than 1.0 to a value near 1.0, independent of 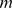 and 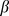. For example, the 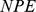 results in original and surrogate data from seven chaotic firings lying between period-1 and period-2 firings are shown in Fig. 7 (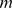 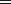
4 and 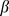 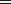
1%), and indicate that all chaotic firings exhibited a short term prediction.

### Reversal sequence of the bifurcation scenario

Readjusting extracellular calcium concentration from 0 mM back to 1.2 mM was conducted in 9 of 22 pacemakers that manifest a bifurcation scenario containing chaotic firing lying between period-1 firing and period-2 firing. A reversal bifurcation sequence was observed in each of 9 pacemakers, containing the pacemaker corresponding to Fig. 4(D) and Fig. 4(E). Readjusting extracellular calcium concentration from 0 mM back to 1.2 mM was not performed on the pacemakers corresponding to Fig. 4(A), Fig.4(C), and Fig. 4(F).

**Figure 8:**
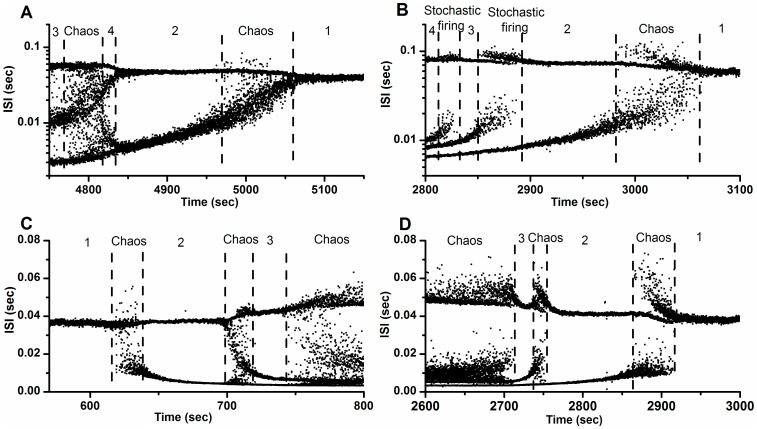
Bifurcation scenarios observed from different neural pacemakers. (A) Inverse bifurcation scenario corresponding to Fig. 4(D) with increasing [Ca^2+^]_o_ from 0 mM to 1.2 mM, whose process was from period-3 firing, to chaotic firing, to period-4 firing, to period-2 firing, to chaotic firing, and to period-1 firing; (B) Inverse bifurcation scenario corresponding to Fig. 4(E) with increasing [Ca^2+^]_o_ from 0 mM to 1.2 mM, whose process was from period-4 firing, to stochastic firing, to period-3 firing, to stochastic firing, to period-2 firing, to chaotic firing, and to period-1 firing; (C) Bifurcation scenario observed in a neural pacemaker with decreasing [Ca^2+^]_o_ from 1.2 mM to 0 mM, whose process was from period-1 firing to period-2 firing via chaotic firing, to chaotic firing, to period-3 firing, and to chaotic firing; (D) Inverse bifurcation scenario corresponding to Fig. 8(C) with increasing [Ca^2+^]_o_ from 0 mM to 1.2 mM, whose process was from chaotic firing, to period-3 firing, to chaotic firing, to period-2 firing, to chaotic firing, and to period-1 firing.

The reversal bifurcation sequence corresponding to Fig. 4(D) was shown in Fig. 8(A). The process of the bifurcations was from period-3 firing, to chaotic firing, to period-4 firing, to period-2 firing, to chaotic firing, and to period-1 firing. The reversal bifurcation sequence corresponding to Fig. 4(E) was depicted in Fig. 8(B). The process was from period-4 firing, to stochastic firing, to period-3 firing, to stochastic firing, to period-2 firing, to chaotic firing, and to period-1 firing.

The bifurcation scenario was observed in a neural pacemaker with decreasing [Ca^2+^]_o_ from 1.2 mM to 0 mM, whose process was from period-1 firing to period-2 firing via chaotic firing, to chaotic firing, to period-3 firing, and to chaotic firing, as illustrated in Fig. 8(C). The inverse bifurcation scenario of this pacemaker with increasing [Ca^2+^]_o_ from 0 mM to 1.2 mM was from chaotic firing, to period-3 firing, to chaotic firing, to period-2 firing, to chaotic firing, and to period-1 firing, as illustrated in Fig. 8(D).

## Discussion

Multiple examples of chaotic firing lying between period-1 firing and period-2 firings observed in the biological experiment performed on isolated neural pacemakers are provided. Most exhibit characteristics similar to those simulated using the HR model. The deterministic mechanism of the chaotic firing and the difference from the stochastic firing are also identified. The results of this experiment and the previously identified two experimental examples [31], [32] demonstrate the existence of the chaotic firing lying between period-1 and period-2 firings in a real nervous system, as simulated by the HR model. The results also show that period-1 firing to period-2 firing via chaotic firing is a novel firing pattern transition process. We hope that this study will enhance interest on the unknown chaotic firing pattern lying between period-1 and period-2 firings. The experimental and theoretical results were obtained from an isolated cell and not from a neuronal network. Bursting synchronization, including chaos synchronization, has been simulated in the networks of coupled HR model neurons, and the role of synaptic coupling in bursting rhythmogenesis has been studied [26]–[28]. The chaotic firing patterns that appear after period-2 firing were the focus of the studies of the network. The chaotic firing that lies between period-1 and period-2 firing patterns and exists in a narrow parameter region needs to be investigated in the neuronal networks in both theoretical and biological models. For example, it is not clear whether this type of chaotic bursting takes place in networks of coupled cells. If it does, then the manner in which endures variations in synaptic coupling raises its own set of questions. Further studies of networks will be able to address this.

Two bifurcation scenarios containing both chaotic firing lying between period-1 and period-2 firings and chaotic firing following period-doubling bifurcation or locating in period-adding sequences, appearing after period-2 firings, were observed in neural pacemakers. These bifurcation scenarios closely match those simulated in the HR model in the parameter space containing two chaotic regions. One is the well-known comb-shaped chaotic region [19]–[25] and the other is the unnoticed chaotic region lying between period-1 and period-2 firings [12]. The two experimental bifurcation scenarios present relationships in parameter space between chaotic firings within the two chaotic regions. In addition, bifurcation scenarios composed of the chaotic firing lying between period-1 and period-2 firings and period-adding sequences with stochastic firings, observed from the fourth example, present the relationships between the chaotic firing lying between period-1 and period-2 firings and the stochastic firings under the comb-shaped chaotic region, similar to the behaviors along the line BL4. Bifurcation scenario containing the chaotic firing lying between period-1 and period-2 firings and period-doubling bifurcation not to chaos, observed from the fifth example and simulated along the line SL1, provides relationships between this chaotic firing and other periodic firing patterns above the chaotic region lying between period-1 and period-2 firings and also right to the comb-shaped chaotic region. These relationships are helpful for the identification of different chaotic, periodic and stochastic firing patterns in the parameter space or within a bifurcation scenario.

The chaotic firing lying between period-1 and period-2 firings of a neural pacemaker can be divided into three parts. Part 1 of the first example, and the previously identified experimental example in ref [31], manifested characteristics different from that of the HR model. On one hand, more experiments should be performed to explore more examples of the chaotic firing lying between period-1 and period-2 firings. On the other hand, some numerical simulation should be performed or reasonable theoretical models should be built to simulate the chaotic firing lying between period-1 and period-2 firings. In addition, the chaotic firing lying between period-1 and period-2 firing can be simulated in multiple parameter spaces of the HR model [19], [24] and observed in the experiment by decreasing [Ca^2+^]_o_ solely or by decreasing [Ca^2+^]_o_ and applying extracellular caesium spontaneously [32]. More physiological parameters should be adjusted in biological experiments to identify the chaotic firing lying between period-1 and period-2 firings and to identify different bifurcation scenarios containing this chaotic firing.

The neural firing patterns were identified play important roles in neural information processing in single electro-sensory afferents of a fish to detect temperature and baroreceptor to regulate blood pressure [1], [2]. The roles of chaotic behaviors observed in different neural systems were discussed [52]. Different firing patterns including the regular period-1 firing and irregular firing patterns were observed at the control condition in the CCI model [39]. One of the mechanisms that could lead to abnormal spontaneous pain, and also contribute to the maintenance of allodynia and hyperalgesia is spontaneous firing of neural pacemaker. The responses of chaotic bursting within a period-adding bifurcation to an external electronic stimulation have been reported and a "critical sensitivity" was proposed to describe the response of the chaotic firing, in contrast to the periodic firing [53]. The spontaneous pain was identified relate to frequency of spontaneous firing [54]. The chaotic firing pattern lying between period-1 and period-2 firing patterns were observed at control condition and were near period-1 firing pattern generated at control condition, which participated the spontaneous pain. Both the HR model and neural pacemakers can exhibit robust chaos in very wide parameter windows, appearing after period-2 bursting and still taking place at the end of the period-doubling or within the period-adding sequences, so that the chaotic firing in the narrower parameter region lying between period-1 and period-2 firing patterns might be much less significant than the ones appearing after period-2 firing from a biological point of view. To investigate the detailed physiological or pathological significance of different firing patterns, including the chaotic firing pattern, of the pacemaker, the animal behaviors such as spontaneous foot lifting, mechanical allodynia and heat hyperalgesia [38], [54] and the electrophysiological signals of neural firing patterns should be recorded simultaneously, which is very difficult to be conducted, awaited to be studied in the future investigation thorough a perfect design of experiment.

The correspondence between the experimental neural pacemakers and the three-dimensional, biologically relevant Chay model, which contains potassium and calcium channels, has been assessed in the previous studies [29], [30], [34]–[36]. The period-doubling bifurcation, period-adding sequences, and bifurcation processes from period-1 bursting to period-1 spiking observed in the neural pacemakers that were induced by changes in the concentration of calcium concentration resembled those simulated using the Chay model, in which these phenomena were induced by adjustment of reversal potential of calcium current. The three-dimensional Sherman model, which shares most of the properties of the Chay model, has a bifurcation process similar to that of the Chay model, as investigated in a previous study [11]. Recently, the dynamics of the Chay model have been investigated in multiple two-dimensional parameter spaces [55], [56]. So far, no chaotic firing pattern lying between period-1 and period-2 firing patterns has been reported in the Chay model or the Sherman model. With the exception of the chaotic firing lying between period-1 and period-2 firing patterns, the firing patterns and bifurcation processes simulated in both the Chay and Sherman models were found to resemble those found in the HR model and of the experimental neural pacemaker. This is why the HR model rather than the Chay or Sherman model was used in the present study. The existence of the chaotic firing patterns lying between period-1 and period-2 burstings needs to be investigated further using a Hodgkin-Huxley (HH)-type model, such as the Chay or Sherman model.
